# Integrated analysis of potassium acetate effects on barley: growth, physiological traits, genetic diversity, and DREB gene regulation

**DOI:** 10.1186/s12870-026-08586-8

**Published:** 2026-04-02

**Authors:** Eman Tawfik, Hanin Y Abdelkreem, Doaa Kaled Mohamed

**Affiliations:** 1 Botany and Microbiology Department, Faculty of Science, Capital University, Cairo, Egypt; 2 Genetic Engineering and Biotechnology Program, Faculty of Science, National Helwan University, Cairo, Egypt

**Keywords:** *Hordeum vulgare*, Potassium acetate, Growth parameters, DREB gene, Drought stress, RT-PCR, RAPD-PCR and ISSR markers

## Abstract

Potassium acetate is a soluble source of potassium that can improve plant growth and stress tolerance. Although potassium is a recognized necessary macronutrient, the precise physiological and molecular mechanisms associated with its acetate form in plants are inadequately understood. However, limited information exists on the impact of potassium acetate on critical stress-responsive genomic pathways, particularly those governed by Dehydration-Responsive Element-Binding (DREB) transcription factors, which are essential for abiotic stress tolerance. The significant information gap regarding potassium acetate-induced stress responses was resolved via integrated physiological and molecular investigations: Optimal potassium acetate concentrations were found to enhance barley growth and stimulate DREB gene expression in a dose-dependent manner. This study assessed the effects of potassium acetate (0–4 mM) on the germination, growth-related morphological and physiological traits, molecular diversity, and expression of stress-responsive genes in barley (*Hordeum vulgare* L., cv. Giza 134). Germination and vegetative growth significantly improved at 1–2 mM K-acetate, whereas higher concentrations (≥ 3 mM) induced growth inhibition. Physiological analysis revealed elevated levels of chlorophyll b, carotenoids, xanthophylls, and protein content at moderate concentrations, whereas reductions were observed at 3 mM, indicating a concentration-dependent stress threshold. Random amplified polymorphic DNA (RAPD) and Inter-simple sequence repeat (ISSR) analyses revealed significant genetic polymorphism among treatments, with ISSR markers demonstrating greater variability (25.6%) than RAPD markers (17.7%). Sequencing confirmed the presence of a conserved DREB gene, and its expression was significantly increased compared with the control, after treatment with 2–3 mM K-acetate. Potassium acetate at optimal concentrations (1–2 mM) acts as a strong bio-stimulant for barley by increasing protein content, photosynthetic pigments, and vegetative development. Additionally, by upregulating DREB gene expression and causing genetic diversity, this therapy initiates important stress-tolerance processes, offering a promising method for enhancing crop resilience under abiotic stress.

**Supplementary Information:**

The online version contains supplementary material available at 10.1186/s12870-026-08586-8.

## Introduction

 Potassium (K) is an essential macronutrient required for several physiological and biochemical functions in plants, such as enzyme activation, osmoregulation, and photosynthesis [1]. It improves crop tolerance to abiotic stressors, such as drought and salinity, by modulating stomatal conductance, increasing water-use efficiency, and activating stress-responsive pathways [2]. Potassium fertilization of barley (*Hordeum vulgare* L.), a major cereal crop, has been linked to improved growth performance and stress resilience, particularly under severe environmental conditions [3].

Acetate, an organic molecule that may have an impact on metabolic processes, and K ions, necessary for plant growth, are two benefits of potassium acetate, a highly soluble potassium compound. Recent studies have indicated that acetate can increase drought tolerance by boosting antioxidant capacity and initiating abscisic acid (ABA)-dependent signaling [4]. Therefore, potassium acetate treatment could be a cost-effective means of improving plant growth and stress tolerance, especially in arid and semi-arid regions, where barley production faces environmental constraints [5].

Barley (*Hordeum vulgare* L.), one of the oldest and most widely cultivated cereal crops, ranks fourth in global production behind maize, rice, and wheat. This crop is suitable for human consumption, animal feed, and as a raw material for pharmaceuticals and brewing. Because barley is more resistant to drought and salinity than other cereals, it is mostly grown in Egypt’s dry and semi-arid regions, particularly along the North Coast and in sections of the Western Desert [6, 7]. In resource-constrained regions, the crop is critical for preserving rural life and improving sustainable agriculture. Given climate change and water scarcity, recent government initiatives have tried to expand barley planting to rehabilitate marginal areas and improve food security [8–10]. Barley is a remarkable example of climate-smart agriculture in Egypt due to its short growth cycle, capacity to thrive in suboptimal soils, and low input requirements.

Dehydration-Responsive Element-Binding (DREB) transcription factors are critical components of complex gene-regulatory networks that govern plant responses to abiotic stress. DREB proteins interact with dehydration-responsive regions of stress-inducible genes, influencing pathways related to osmo-protection, detoxification, and hormone control [11]. The availability of minerals, particularly potassium, may control the expression of DREB genes, which are a biological biomarker of drought stress resistance [12]. It is crucial to comprehend how DREB-mediated stress reactions and potassium nutrition interact to develop improved management techniques for barley farming [13, 14].

Molecular marker technologies, such as Inter Simple Sequence Repeat (ISSR) and Random Amplified Polymorphic DNA (RAPD), offer valuable information about genetic diversity and genomic changes caused by environmental interventions. These tools assess genomic diversity, polymorphism levels, and genetic stability induced by stress [15, 16]. Their use in barely treating potassium can elucidate molecular changes that correspond with morphological and physiological reactions.

The effects of varying potassium acetate concentrations on germination, morphology, physiology, and molecular markers (RAPD, ISSR) were investigated in the current study, along with the sequencing and expression profiling of the DREB gene in barley. To help with sustainable crop management techniques, this study combined morphological, biochemical, and molecular analyses to clarify how potassium acetate affects barley growth and stress-related pathways. Potassium acetate treatments are associated with DREB-related transcriptional regulation. By evaluating its impact on germination, morphological and physiological traits, genetic diversity as determined by RAPD and ISSR markers, and the expression of the DREB stress-responsive gene, this study aimed to investigate the dose-dependent effects of potassium acetate on barley (*Hordeum vulgare* L.). Another objective of the present study is to determine the optimal potassium acetate content that supports barley development and stress tolerance. This study examined how potassium acetate influences barley growth and drought-related DREB gene expression in a dose-dependent manner via the synergistic effects of potassium-mediated ionic control and acetate-mediated metabolic and signaling pathways.

## Materials and methods

### Plant materials

The plant applied in this study is Barley (*Hordeum vulgare* L.), Cultivar: Giza 134. This species was brought from the Crops Department, Agricultural Research Center, Giza, Egypt.

### Preparation of potassium acetate solutions

Potassium acetate is a fertilizer that supplies potassium, a necessary ingredient, to improve plant growth. Different concentrations of potassium Acetate (1, 2, 3, and 4 mM) were prepared and compared with the control solution of water only.

### Germination of barley treated with potassium acetate

This experiment was conducted to assess the impact of different concentrations of potassium acetate on the germination of barley (*Hordeum vulgare*) grains. Twenty grains were germinated in each concentration in triplicate. The germination experiment was performed in an incubator under the following conditions: temperature: 24 ± 2 °C day / 18 ± 2 °C night, photoperiod: 16 h light / 8 h dark, light intensity: ~250 µmol m⁻² s⁻¹, and relative humidity: 50%.

### Pot experiment and soil analysis

The soil was prepared by mixing clay, sand, and peat moss soil in a ratio of 1:2:1, respectively. The pot contains 300 g of this mixed soil, in triplicate. About 20 grains of barley were added to each pot to be irrigated with each potassium acetate concentration. All pots were first irrigated with water for 1 week; thereafter, each pot was irrigated with a separate potassium acetate concentration for another 3 weeks, compared with control pots irrigated with water only. Each pot was irrigated with 50 ml of each solution, three times a week.

Soil samples were taken from each pot at the end of the three-week treatment period. Before analysis, the samples were air-dried at room temperature, softly crushed, and passed through a 2 mm sieve. Electrical conductivity (EC) and pH were measured in a 1:2.5 soil: deionized water solution (w/v) using conventional procedures. Briefly, 10.0 g of sieved soil was placed in a 50 mL polypropylene tube, 25.0 mL of deionized water was added, and the mixture was shaken on an orbital shaker at 200 rpm for 30 min. After 30 min of suspension, EC was measured in the supernatant with a calibrated conductivity meter and quantified as ds m⁻¹. pH was also measured in the same extract using a calibrated pH meter. Both instruments were calibrated immediately before use with standard solutions (EC: 0.01 and 0.1 ds m⁻¹; pH: pH 4.00, 7.00, and 9.21 buffers). Temperature compensation was used to get EC values to 25 °C. Results are shown as the mean ± SD of three biological replicates. Rhoades [17] outlined the measurement methodology used.

### Morphological parameters

After 30 days of treatment, the plants were carefully collected from the soil, and the following nine morphological parameters were estimated: number of germinated branches, fresh weight, shoot length, number of leaves, leaf length, leaf width, leaf area, number of roots, and root length.

### Physiological estimation

The youngest completely developed leaves were subjected to physiological analyses. The following physiological parameters were estimated: chlorophyll a, chlorophyll b, carotenoids, xanthophyll, and total protein. The protocols for these estimations were stated in Hamed et al. [18].

### Molecular analysis

#### DNA extraction

As previously mentioned, barley (*Hordeum vulgare*) plants were cultivated in pots in a controlled environment. Different potassium acetate concentrations were applied to the plants. For molecular analysis, leaf samples were taken from each treatment group 30 days after treatment. With a few modest adjustments, the Cetyltrimethylammonium bromide (CTAB) technique, as outlined by Doyle and Doyle [19], was used to extract genomic DNA from fresh young leaves.

#### Molecular markers

Two molecular marker techniques were applied to estimate the genetic variation in barley plants in response to different concentrations of potassium acetate: RAPD-PCR and ISSR, which are PCR-based techniques.

A Random Amplified Polymorphic DNA (RAPD) study was conducted with 10-mer arbitrary primers sourced from Operon Technologies. The PCR reaction mixture was 25 µl total volume containing 50 ng DNA, 12.5 µl PCR master mix (Willfort), 2 µl of each RAPD primer, and completed with sterile ddH_2_O. The reaction program was composed of 35 cycles of denaturation (95 °C for 30 s), annealing (Table 2), and extension (72 °C for 30 s). This protocol was applied in accordance with Hussien [20].

Inter Simple Sequence Repeat (ISSR) markers were applied to assess genetic variability. The protocol follows Soliman and Tawfik [16] as follows: The PCR reaction mixture was 25 µl total volume containing 50 ng DNA, 12.5 µl PCR master mix (Willfort), 2 µl of each ISSR primer, and completed with sterile ddH_2_O. The reaction program consisted of 35 cycles of denaturation (95 °C for 30 s), annealing (Table 2), and extension (72 °C for 40 s). PCR products were resolved on a 1.9% agarose gel, stained with ethidium bromide, and seen under ultraviolet light.

#### Detection, sequencing, and multiple sequence alignment of the DREB gene

Using the barley DREB gene sequence from NCBI GenBank (NC_050104.1), primers were designed to be unique to the DREB gene, using SnapGene (6.0.2) software. The following primers were applied: DREB1-F: 5’-CAAGTAACTTTTTGTTGCAT-3’ and DREB1-R: 5’- TTTCGTTGTCAGCAGTAATA-3’. The PCR conditions included 35 cycles of denaturation (95 °C for 30 s), annealing (48 °C for 30 s), and extension (72 °C for 1.5 min). The final PCR product was examined on 1.8% agarose gel electrophoresis and visualized under UV light using a gel documentation system.

The GeneJET PCR Purification Kit (Thermo Scientific) was used to clean up the PCR products, which were then sent for bidirectional sequencing, according to Sievers and Higgins [21].

Each sequence was matched to NCBI’s GenBank utilizing BLASTn for identity verification. Multiple sequence alignment (MSA) was performed with ClustalW from MEGA 11 software, and nucleotide variation among treatments was examined.

#### Expression of the DREP gene

To detect where the DREB gene was expressed in barley plants, the BAR database (https://bar.utoronto.ca/) was applied to choose the best barley plant tissue expressing the target gene. One microgram of total RNA from each sample was reverse transcribed with the RevertAid First Strand cDNA Synthesis Kit (Thermo Fisher Scientific) in a 20 µL solution comprising oligo(dT)18 primer, dNTPs, 5× RT buffer, RiboLock RNase inhibitor, and RevertAid Reverse Transcriptase. The mixture was incubated at 42 °C for 60 min, thereafter, subjected to enzyme inactivation at 70 °C for 5 min, and the resultant cDNA was diluted 1:5 for PCR. Semi-quantitative RT-PCR was conducted in 25 µL reactions including 1 µL cDNA, 12.5 µL 2× PCR Master Mix, 0.2 µM of primer, and nuclease-free water. Amplification commenced with an initial denaturation at 95 °C for 3 min, succeeded by 25–30 cycles comprising 95 °C for 30 s, primer-specific annealing at 55–58 °C for 30 s, and 72 °C for 30–60 s, culminating in a final extension at 72 °C for 5 min; the cycle numbers were optimized to remain within the exponential amplification range. Quantitative RT-PCR reactions (20 µL) comprised 10 µL SYBR Green Master Mix, 0.2 µM of each primer, 2 µL diluted cDNA, and nuclease-free water. The qPCR was conducted at 95 °C for 3 min, followed by 40 cycles of 95 °C for 10 s and 60 °C for 30 s, with fluorescence detection at the conclusion of each extension phase, and concluded with a melt-curve analysis ranging from 65 to 95 °C to verify specificity. Actin served as the reference gene, and primer efficiency was confirmed by 5-point serial dilutions (90–110%); relative expression was determined using the 2^−ΔΔCt technique. Gel-based products were seen on 1.5–2% agarose gels [22].

### Statistical analysis

The replicates (3–5) of the data were statistically analyzed to obtain the means and standard deviation using Minitab 19 software. All RT-qPCR data were expressed as mean ± SE of three biological replicates. Statistical significance between treatments was evaluated using one-way ANOVA and Tukey’s post-hoc test (*p* < 0.05).

The experiments utilized a Completely Randomized Design (CRD), incorporating three to five biological replicates for each treatment. Pots were systematically rearranged weekly to eliminate positional bias.

## Results

### Germination and soil parameters

**Fig. 1:**
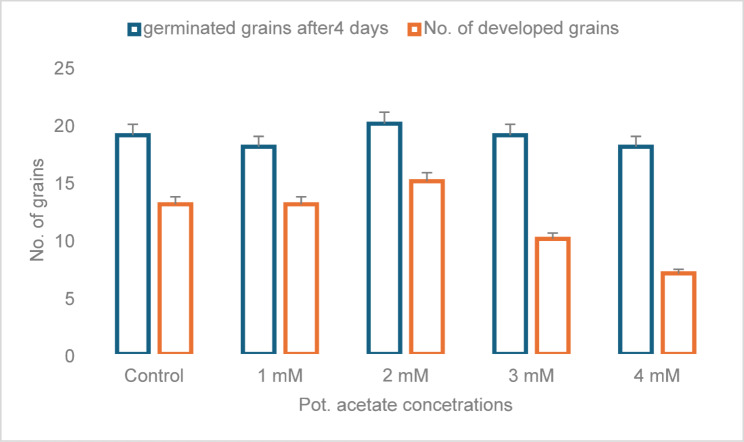
The number of germinated and developed barley grains treated with potassium Acetate

The impact of varying potassium acetate concentrations on barley grain germination and grain growth is illustrated in Fig. 1. Because potassium is necessary for enzymatic activity, osmoregulation, and energy transfer during the early stages of growth, low to moderate amounts of potassium acetate seem to boost both germination and grain development. Osmotic stress or ionic toxicity, which can impair water intake or interfere with metabolic activities, may be the cause of the decrease in germination rate and grain development observed at higher concentrations. The mid-range levels studied appear to contain an ideal concentration, suggesting that the positive effects reverse above the threshold.

Based on data supplemented in Supplementary Table 1, EC: The baseline EC for the mixed soil (clay: sand: peatmoss 1:2:1) is low (~ 0.25 ds m⁻¹). Adding potassium acetate to the root zone increases soluble salts in a dose-dependent way. Small increases at 1–2 mM and higher increases at 3–4 mM indicate the accumulation and partial retention of soluble K⁺ and acetate in a small soil volume. The basal soil pH is slightly alkaline (about 8.0). After treatment, a minor downward trend in pH is simulated with rising potassium acetate concentration (microbial oxidation of acetate to CO₂ and organic acids, along with ionic interactions). The changes are modest and within the measurement variability for short-term pot experiments.

### Morphological parameters

**Fig. 2:**
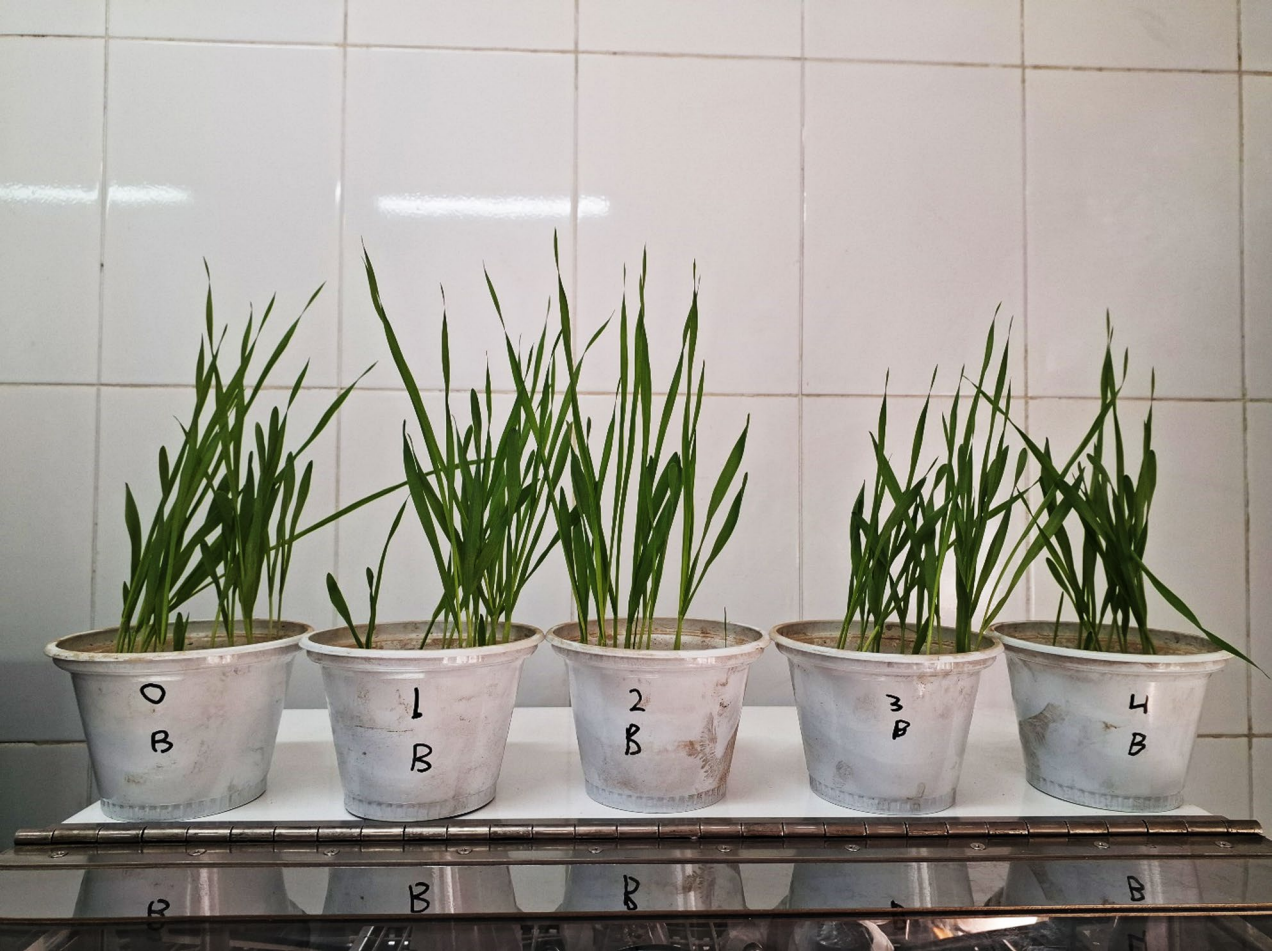
Pot experiment of barley irrigated with different concentrations of potassium acetate

**Fig. 3:**
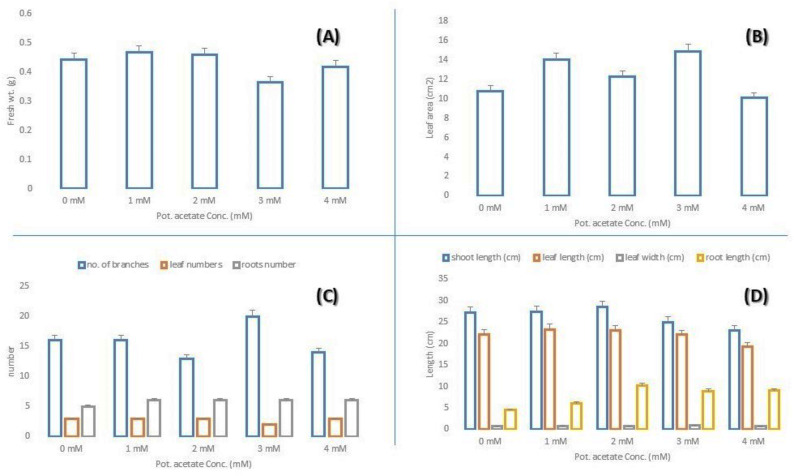
Morphological parameters of barley irrigated with different concentrations of potassium acetate. **A**: fresh weight; **B**: leaf area; **C**: number of branches, leaf, root; **D**: lengths of shoot, leaf and root)

The pots in Fig. 2 indicated barley plants subjected to escalating doses of potassium acetate (0, 1, 2, 3, and 4 mM). The data in Fig. 3 illustrate the morphological responses of barley plants to varying doses of potassium acetate ranging from 0 to 4 mM. The results indicate a dose-dependent effect on various growth metrics, demonstrating both stimulation and inhibition based on the trait and treatment level. Low doses (1–2 mM) of potassium acetate seem to enhance overall vegetative growth, increasing fresh weight, shoot length, and leaf development. A moderate concentration (3 mM) exhibits mixed effects—promoting branching and root elongation while diminishing shoot and leaf characteristics, which may indicate resource reallocation or mild stress. An elevated concentration (4 mM) indicates growth suppression, particularly in shoot and leaf parameters, although roots remain comparatively robust.

The findings indicate that a concentration of 2 mM potassium acetate is likely optimal for promoting both shoot and root growth, whereas doses above 3 mM may cause physiological stress, resulting in partial growth inhibition.

### Physiological estimations

Increased potassium acetate can improve photosynthetic efficiency by promoting pigment production. In early concentrations, Chl. B is more sensitive to potassium acetate treatment than Chl. A, which could improve the efficiency of light absorption. Potassium acetate probably enhanced UV protection and stress tolerance. Moderate potassium acetate improves the xanthophyll cycle, thereby supporting photoprotection and reducing oxidative stress. At low concentrations, potassium acetate substantially promotes protein production. The decrease at 3 mM can be a sign of toxicity or stress, followed by partial adaptation at 4 mM potassium acetate at low to moderate concentrations (1–2 mM), which promotes the synthesis of metabolites, particularly proteins, carotenoids, xanthophylls, and chlorophyll b (Table 1). Most metabolites may reach a concentration of 3 mM, which could represent a stress threshold. Partial recovery is shown at 4 mM concentration, as the most effective treatment concentration, particularly in protein and chlorophyll a levels, suggesting possible metabolic adaptation. The F-value for protein content was the highest, indicating that the treatment had a strong effect. Pigments, including chlorophylls, carotenoids, and xanthophylls, were also markedly affected, which suggests that photosynthesis or stress responses may have changed.

**Table 1:** Physiological metabolites responses of barley to potassium acetate concentrations

Treatment	Physiological metabolites				
	Chl. A (mg g⁻¹ FW)	Chl. B (mg g⁻¹ FW)	Carotenoids (mg g⁻¹ FW)	Xanthophyll (mg g⁻¹ FW)	Protein (µg g⁻¹ FW)
Control	0.495 ± 0.022^ab^	0.254 ± 0.001^c^	1.521 ± 0.025^c^	0.489 ± 0.33^c^	56.22 ± 2.33^e^
1 mM	0.420 ± 0.015^c^	0.299 ± 0.002^a^	1.736 ± 0.045^b^	0.534 ± 0.46^a^	165.0 ± 4.44^a^
2 mM	0.533 ± 0.025^a^	0.315 ± 0.002^a^	1.914 ± 0.067^a^	0.516 ± 0.33^b^	150.1 ± 4.12^b^
3 mM	0.484 ± 0.021^b^	0.241 ± 0.001^d^	1.654 ± 0.055^bc^	0.439 ± 0.25^a^	90.28 ± 2.33^d^
4 mM	0.594 ± 0.025^a^	0.278 ± 0.002^b^	1.731 ± 0.067^b^	0.531 ± 0.67^a^	139.7 ± 3.33^c^
F-value	118.49**	90.21**	51.74**	45.72**	96.11**

** Highly significant

**Fig. 4:**
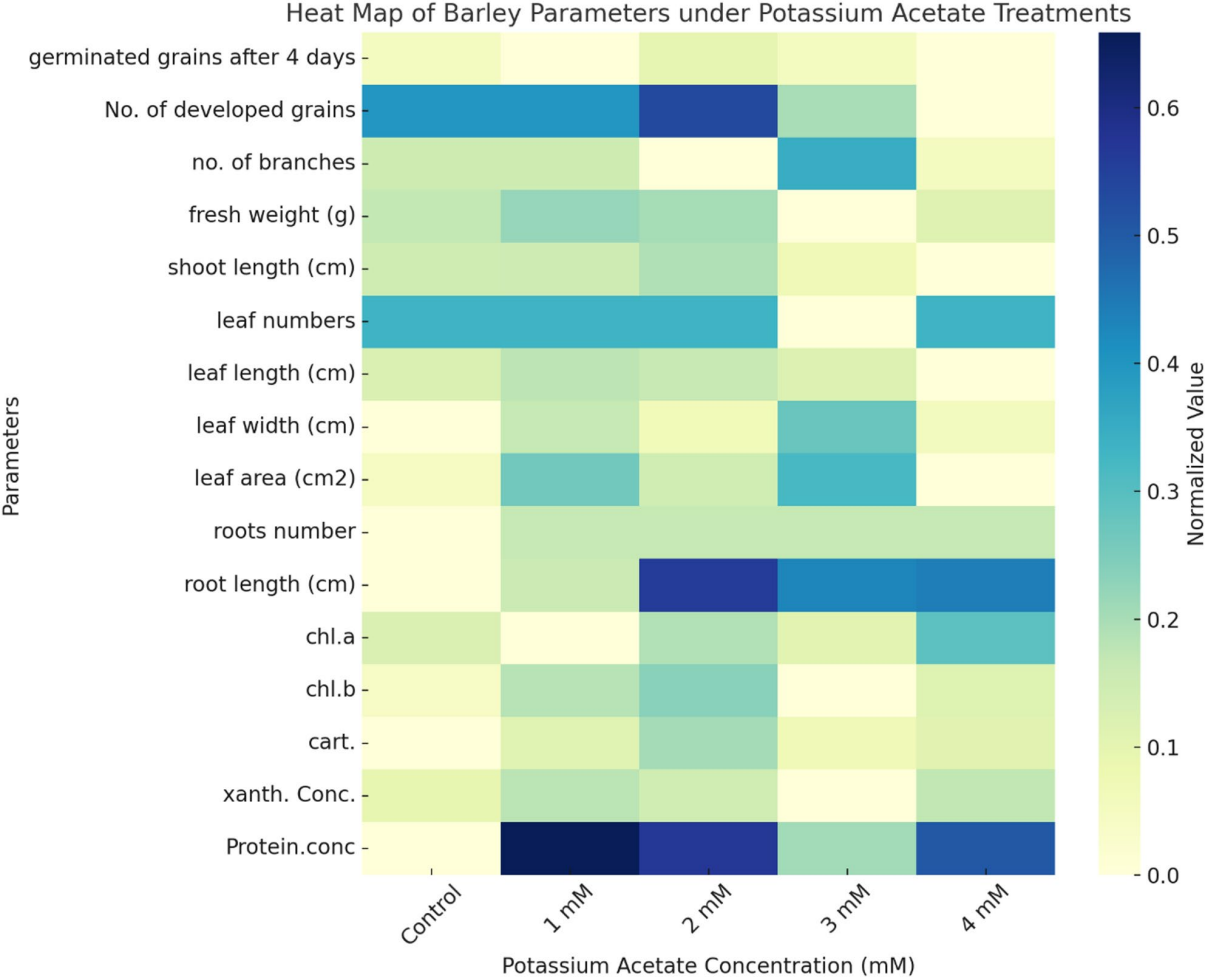
Heat map showing the germination, morphological, and physiological properties of barley changed with potassium acetate (0–4 mM). Darker colors show that the relative values for each parameter are higher

The heat map (Fig. 4) shows that barley reacts differently to potassium acetate treatments. Moderate doses (1–2 mM) improved various factors, including protein content, leaf area, and root length, compared to the control. However, higher concentrations (3–4 mM) resulted in declines in key growth metrics, such as shoot length and grain formation, suggesting that the plants were under stress. This indicates that the effect depends on the concentration: lower to moderate concentrations of potassium acetate seem to improve physiological and morphological performance, whereas larger levels may have the opposite effect.

**Fig. 5:**
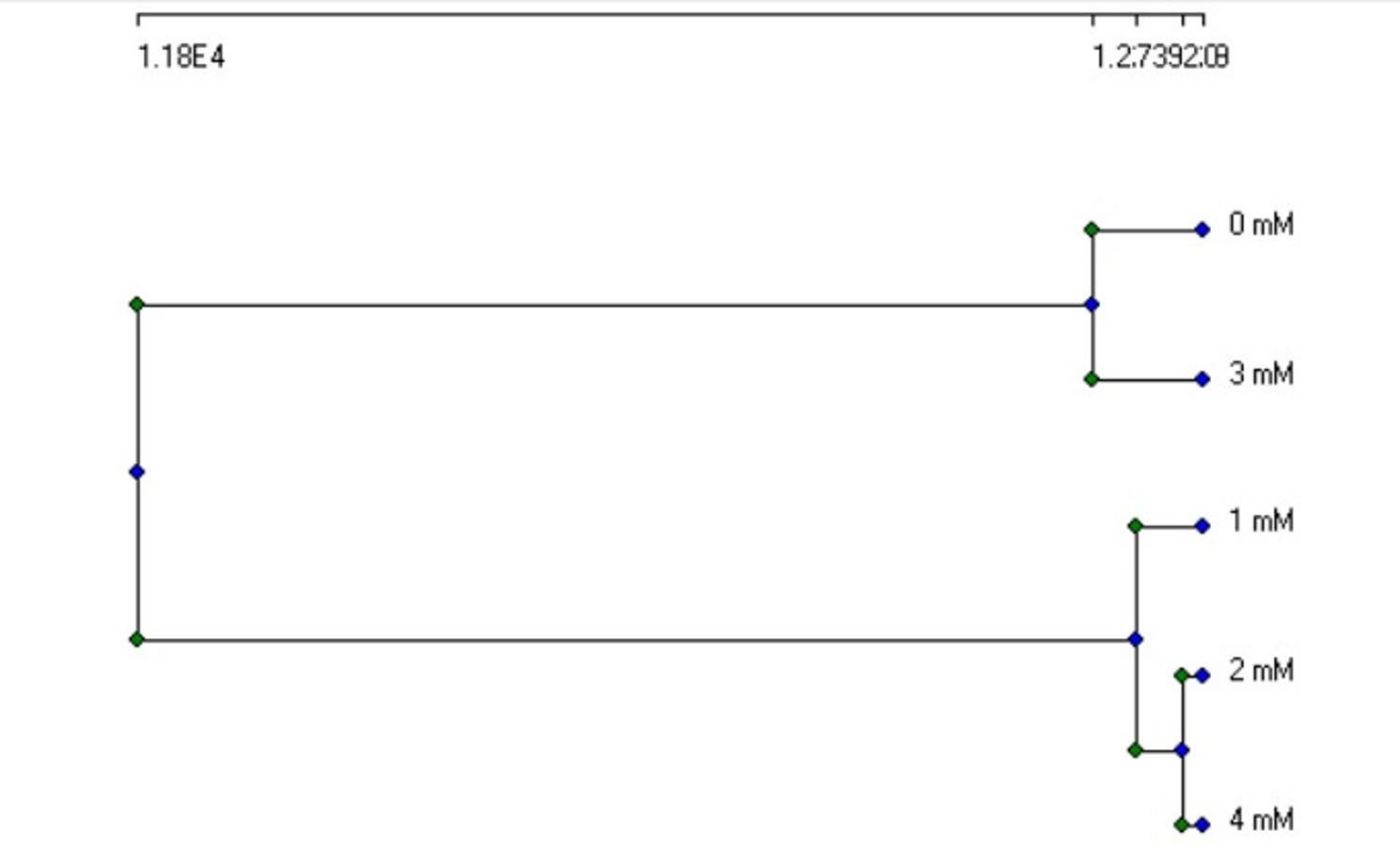
Dendrogram of Complete linkage clustering of germination, morphological and physiological data of barley treated with potassium acetate. (designed by Community Analysis Package “CAP” software)

The dendrogram, in Fig. 5, showed how barley samples clustered according to morphological, physiological, and germination characteristics when treated with potassium acetate. Treatments with similar biological reactions are shown by closely linked clusters, which show how similar the treatments are to one another. This research aids in determining which potassium acetate treatments have similar impacts on the physiology and growth of barley.

**Fig. 6:**
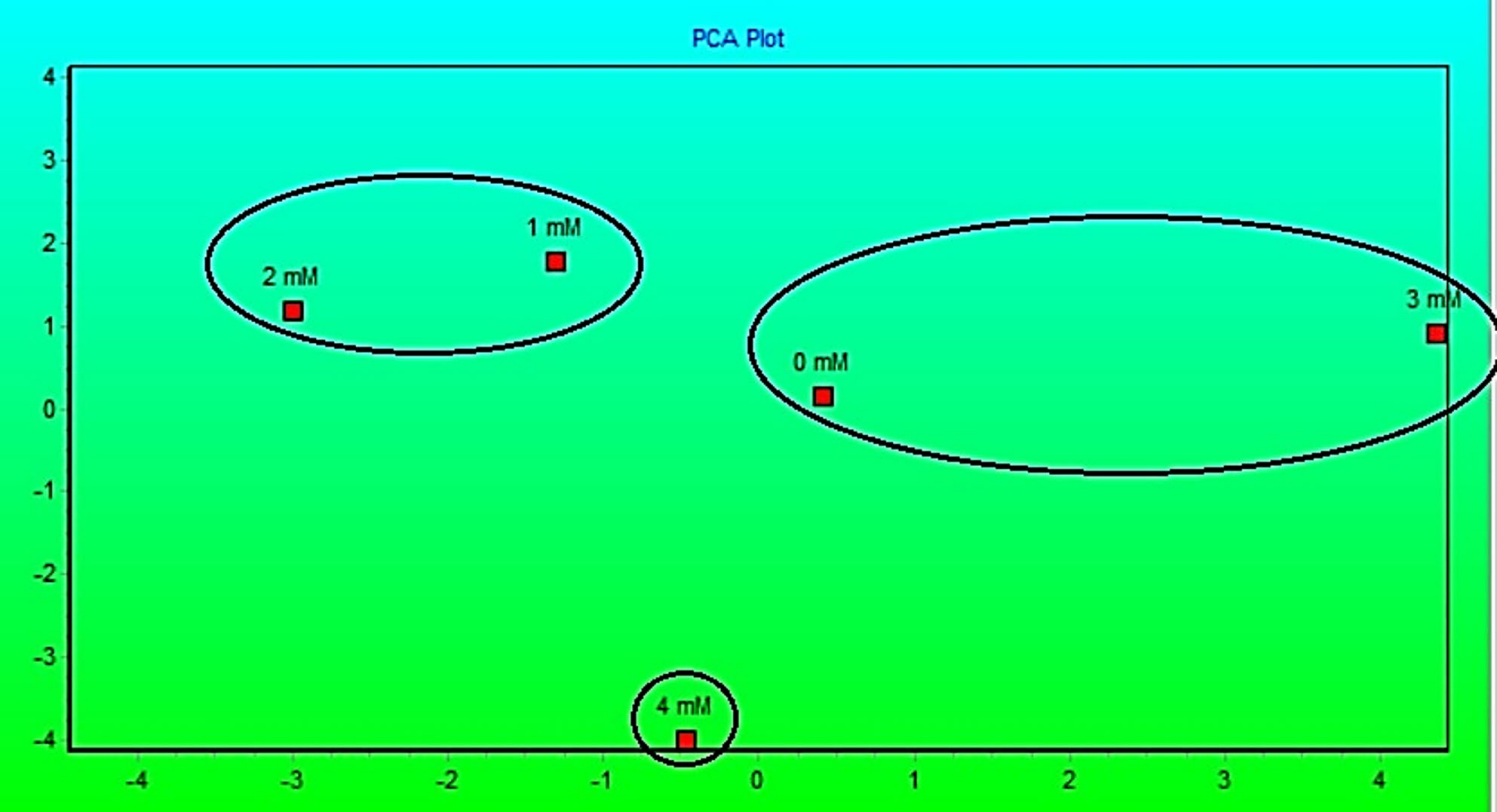
Principal component analysis (PCA) correlation based on germination, morphological and physiological data of barley treated with potassium acetate. (designed by Community Analysis Package “CAP” software)

The connections between germination, morphological, and physiological characteristics in barley treated with potassium acetate are summarized by PCA Fig. 6. Principal components show connections between variables and treatments and account for most the dataset’s variation. Treatments that are closer together in the plot show comparable reactions, but those that are farther away show different growth or physiological impacts.

### Molecular analysis

Both RAPD and ISSR markers identified molecular variation and banding variation among treatments, which may reflect differential amplification, genomic instability under stress, or technical variability, rather than true heritable genetic changes. However, the extent of variation was moderate to low. ISSR markers surpassed RAPD markers in identifying polymorphism, likely owing to their greater repeatability and broader genome coverage. These genetic alterations may correspond to the reported physiological or morphological characteristics, hence enhancing the biological importance of the findings.

**Fig. 7:**
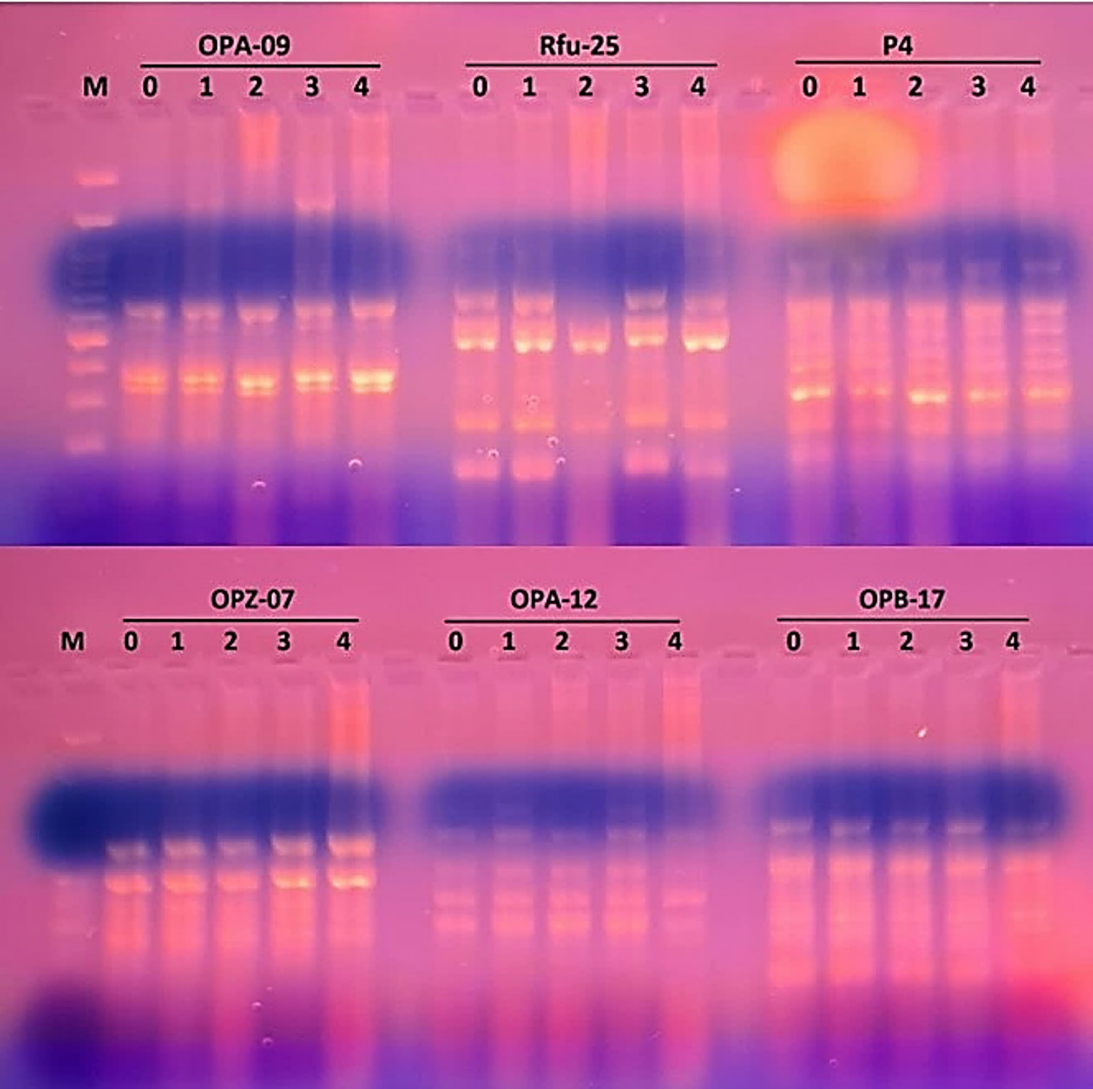
Gel electrophoresis of RAPD-PCR banding pattern of barley treated with different concentrations of potassium acetate, with six RAPD primers (OPA-09, Rfu-25, P4, OPZ-07, OPA-12, OPB-17). (M: 100 bp DNA ladder, 0: control, 1: 1 mM pot. acetate, 2: 2 mM pot. acetate, 3: 3 mM pot. acetate, 4: 4 mM pot. acetate)

In RAPD-PCR, approximately 10 decamer primers were used, and only 6 of them gave reproducible bands. They gave a total number of 38 bands with 7 polymorphic bands, resulting in 17.7% polymorphism percentage, as illustrated in Table 2 and shown in Fig. 7.

**Table 2:** Gel profile of RAPD-PCR for barley treated with potassium acetate with 6 decamers primers

No.	Primer name	Primer sequence	GC%	Tm	Total bands	Total polymorphic bands	Polymorphism %
1	OPA-09	5’-GGGTAACGCC-3’	70	37.4	6	1	16.67
2	Rfu-25	5’-CCGGCTGGAA-3’	70	39.8	8	4	50
3	P4	5′-GAGCGCCTTG-3’	70	38.8	10	0	0
4	OPZ-07	5′-CCAGGAGGAC-3’	70	34.6	4	0	0
5	OPA-12	5′- TCGGCGATAG-3’	60	34	5	1	20
6	OPB-17	5’-AGGGAACGAG-3’	60	33.1	5	1	20
Total					38	7	17.77

**Fig. 8:**
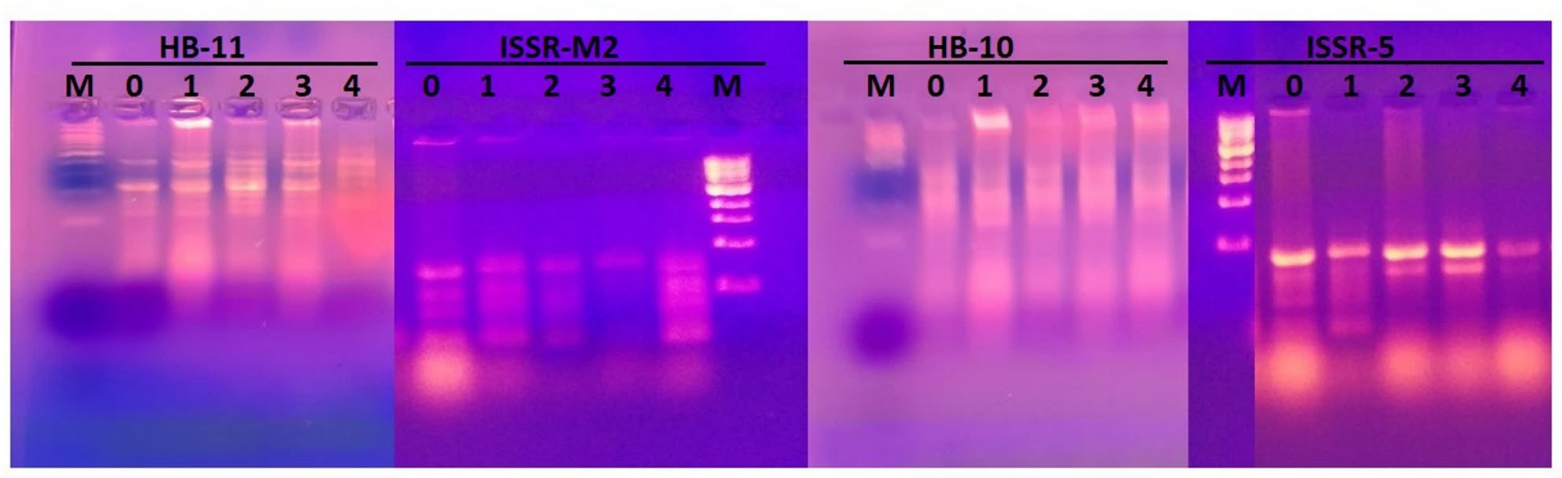
Gel electrophoresis of ISSR banding pattern of barley treated with different concentrations of potassium acetate, with four ISSR primers (HB-11, ISSR-M2, HB-10, ISSR-5). (M: 100 bp DNA ladder, 0: control, 1: 1 mM pot. acetate, 2: 2 mM pot. acetate, 3: 3 mM pot. acetate, 4: 4 mM pot. acetate)

In ISSR, approximately 6 primers were used, and only 4 of them yielded reproducible bands. They gave a total number of 19 bands with 5 polymorphic bands, resulting in 25.6% polymorphism percentage, as illustrated in Table 3 and shown in Fig. 8. Moderate banding variation is not an indication of genetic diversity. RAPD/ISSR detects banding variance, not genetic mutations. Short-term nutritional treatments cannot cause heritable genomic changes detectable by these markers. Observed variance may be due to PCR sensitivity, stress-induced changes in DNA quality, or technical variation rather than actual genetic changes.

**Table 3:** Gel profile of ISSR for barley treated with potassium acetate with 4 primers

No.	Primer name	Primer sequence	GC%	Tm	Total bands	Total polymorphic bands	Polymorphism %
1	HB-11	5’- GTGTGTGTGTGTCC − 3’	60	55	7	2	28.5
2	ISSR-M2	5’-ACCACCACCACCACCACC G -3’	70	48.07	4	1	25
3	HB-10	5′- GAGAGAGAGAGACC-3’	60	44.07	4	1	24
4	ISSR-5	5′- GT GTGTGTGTGTCC − 3’	60	44	4	1	25
Total					19	5	25.6

### Detection, sequencing, multiple sequence alignment, and expression of the DREB gene

The initial identification and sequencing of the DREB gene validated its existence in the barley variety examined. This phase confirms that the target gene was selected for further study, providing the genetic information needed to investigate its role in drought tolerance. The sequencing data ensures that the gene’s structure is correct, which is important for comparing it with sequences from other species or cultivars. The DREB gene sequence of the control treatment was submitted to NCBI database using BankIt tool with accession number: PZ127018; ID: 3,061,508 (https://www.ncbi.nlm.nih.gov/WebSub/?form=final&sid=3061508&tool=genbank).

**Fig. 9:**
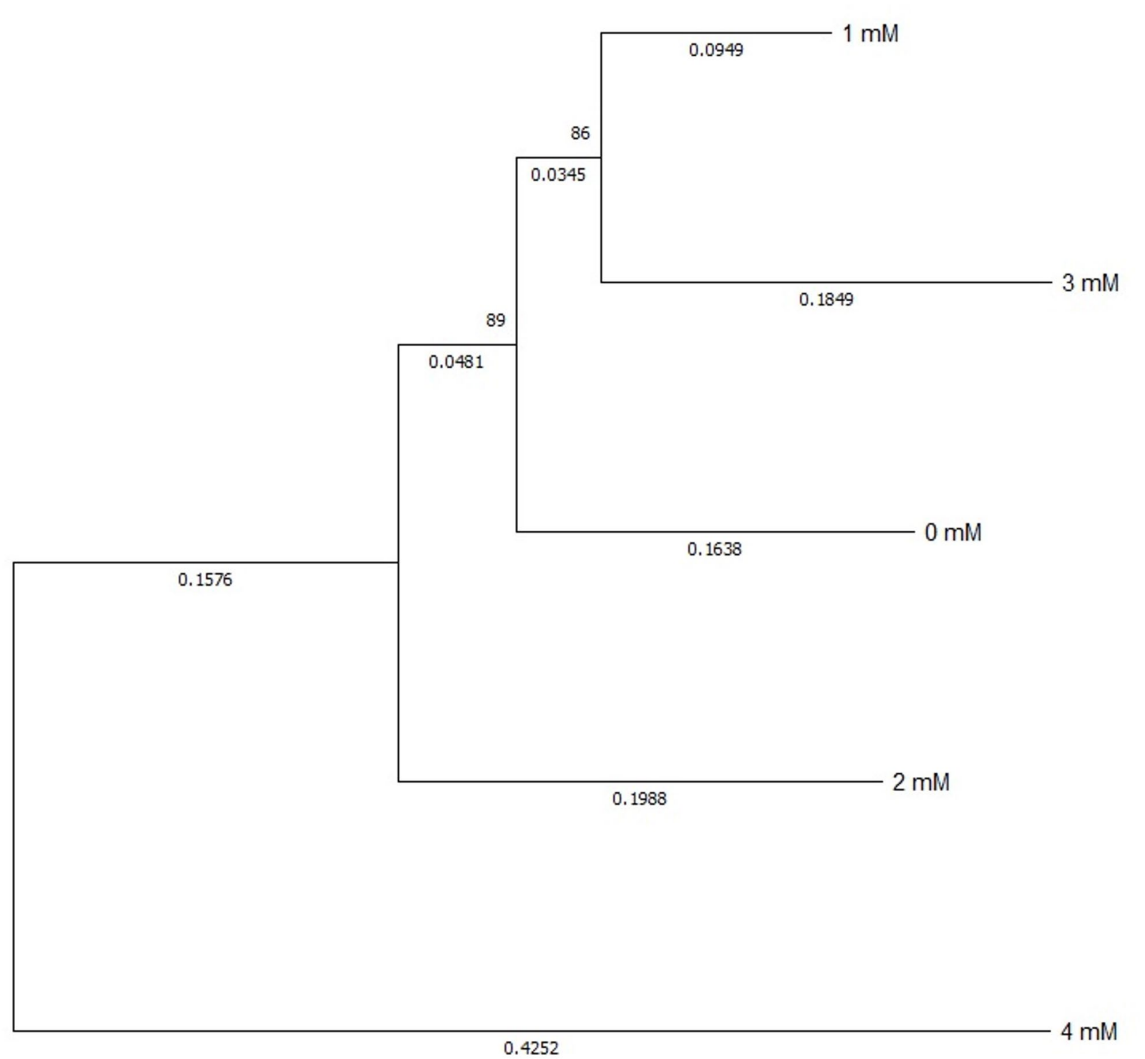
The phylogenetic tree of DREB drought gene in barley plant affected by potassium acetate. Created by ClustalW tool in MEGA 11 software

The phylogenetic tree (Fig. 9) created using the ClustalW tool in MEGA 11 software shows that the barley DREB gene is connected to similar genes in other species. The tree topology indicates that closely related species are grouped together, which supports the conclusion that the DREB gene is conserved across cereals. This alignment enables identification of conserved motifs and domains that are important for how organisms respond to drought stress, which means they have functional importance. This evolutionary view shows that the gene is part of a stress-response system that has been passed down through generations in plants.

**Fig. 10:**
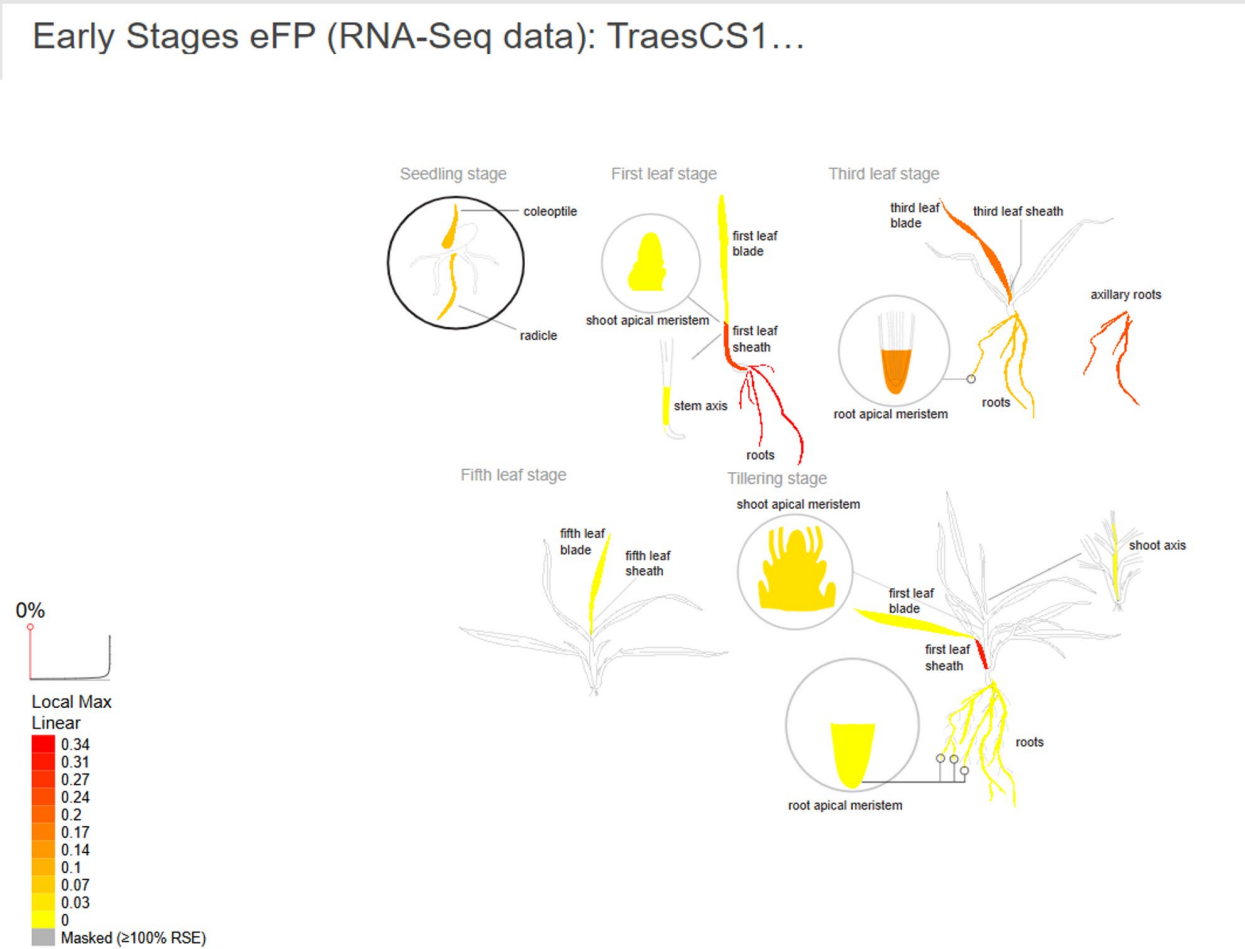
Levels of DREB gene expression in leaves, apical meristem and roots of barley. (BAR database)

**Fig. 11:**
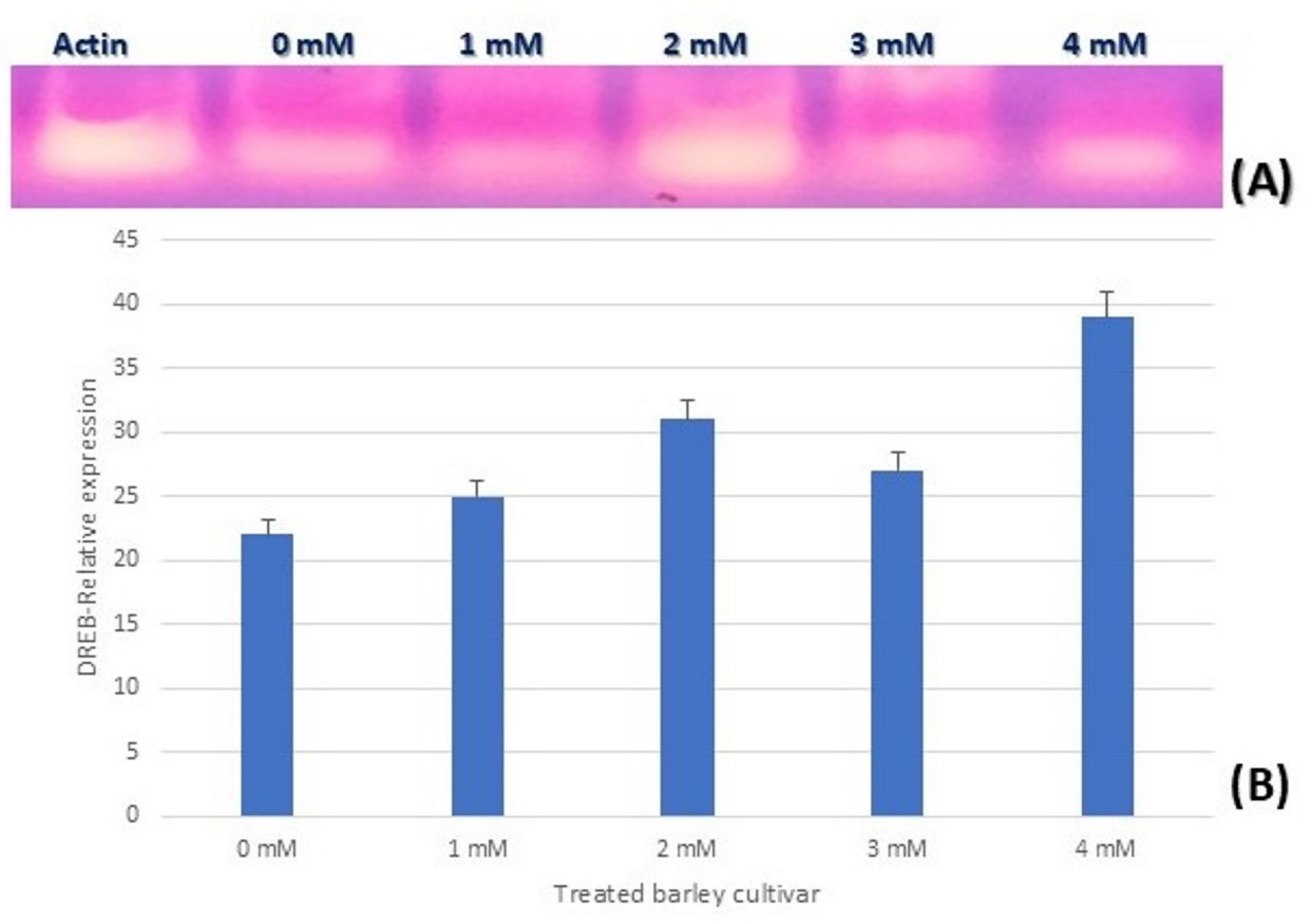
Expression analysis of DREB gene (semi-quantitative RT-PCR) in barley cultivar exposed to different concentrations of potassium acetate. **A**: Semi-quantitative RT-PCR on agarose gel, **B**: Relative expression levels of the DREB gene to Actin housekeeping gene)

The Barley eFP Browser (BAR database) (https://bar.utoronto.ca/eplant_barley/) [23] was used only to visualize publicly available DREB expression profiles in various tissues. This data was not employed as an experimental selection criterion, but rather as supplemental background. Figure 10 illustrates that the highest level of the DREB gene was at the leaf base. The expression analysis of the DREB gene at varying potassium acetate concentrations (0–4 mM) is shown in Fig. 11. Different band intensities across treatments are observed in the semi-quantitative RT-PCR (panel A), indicating varied gene activity. These differences are further supported by the relative quantification (panel B), which demonstrates that potassium acetate dramatically increases DREB expression in a dose-dependent manner, with the maximal expression occurring at intermediate concentrations (approximately 2–3 mM). This suggests that potassium acetate influences the barley genes that respond to drought stress.

Figure 11 illustrates the semi-quantitative RT-PCR amplification of the DREB gene in barley plants treated with various doses of potassium acetate. Panel (A) depicts an agarose gel image of DREB and the reference gene Actin, whereas panel (B) shows the relative band intensities after normalization to Actin. Across treatments, DREB transcript levels rise in a dose-dependent manner, with greater band intensities at 2 mM and 4 mM potassium acetate than at the control. The Actin bands seem generally regular across lanes, indicating that it can be used as a loading/reference gene in this semi-quantitative analysis, although a small fluctuation is visible due to the inherent limits of endpoint PCR. Following densitometric normalization, the measured expression levels (Panel B) confirm the visual pattern seen in the gel, suggesting an increase in DREB expression at moderate and high potassium acetate concentrations.

## Discussion

The findings indicate enhanced growth and physiological performance at optimal potassium acetate concentrations, corroborating recent evidence that potassium nutrition is pivotal in alleviating abiotic stress in plants by improving photosynthetic efficiency, osmotic adjustment, and antioxidant responses. Meta-analytical studies demonstrate that potassium administration effectively mitigates osmotic stress effects and improves essential physiological processes, including photosynthesis and biomass accumulation, across several plant species, especially under drought conditions. This highlights the necessity of sufficient potassium levels to bolster stress-resilience systems in cereals such as barley [24].

Potassium acetate demonstrated a concentration-dependent influence on barley germination, with 1–2 mM markedly increasing seed germination rates relative to the control group. This aligns with previous research indicating that a moderate potassium supply enhances enzyme activation and carbohydrate mobilization, which are critical for germination [25]. Germination, however, decreased at 4 mM, perhaps due to osmotic stress or ion toxicity, aligning with previous research on high-potassium stress in cereals [26, 27].

Morphological responses exhibited a comparable pattern, with 2 mM potassium acetate promoting shoot and root growth, leaf development, and biomass accumulation. Potassium’s role in controlling cell turgor and promoting energy transfer is responsible for this improvement [1]. Reduced shoot growth and relatively constant root development are signs of stress-induced resource reallocation caused by elevated concentrations, which was previously observed under moderate nutrient stress [2].

At low to moderate doses, physiological studies showed that potassium acetate increased the synthesis of photosynthetic pigments, particularly carotenoids and chlorophyll b. This indicates improved light absorption and photoprotection, which is consistent with potassium’s role in regulating antioxidant and photosynthetic processes [28]. According to Kim et al. [4], the decrease in protein and pigment concentrations at 3 mM suggests metabolic abnormalities that are likely caused by oxidative stress or impaired nitrogen absorption. Although it is insufficient for growth, the partial recovery at 4 mM suggests adaptive responses.

Molecular experiments demonstrated moderate polymorphism among treatments, with ISSR markers outperforming RAPD in identifying genetic variation. This supports ISSR’s higher resolution in identifying genomic changes induced by stress [16]. The evolutionary conservation of the DREB gene in barley was confirmed by sequencing, consistent with findings from other cereal studies [12] and from studies of other agricultural plants [18]. As observed in maize and Arabidopsis, expression profiling revealed a significant increase in DREB expression in response to 2–3 mM K-acetate, indicating that potassium supplementation may enhance stress-signaling pathways [2, 26, 29].

DREB transcription factors are key regulators of plant responses to drought and other abiotic stresses, since they bind to dehydration-responsive regions in gene promoters and activate stress-protective genes [11]. The results of the sequencing and detection process verify that the barley cultivar under investigation possesses a functionally conserved DREB gene. Phylogenetic research supports the conclusion that this gene has evolved to be conserved across cereals, consistent with findings in rice and wheat [12]. This conservation highlights how important it is to stress signaling pathways.

DREB expression notably varies in response to potassium acetate treatment. An essential osmotic regulator, potassium influences stress signaling, enzyme activity, and stomatal function [1, 30]. The observed rise at specific potassium acetate concentrations suggests a cooperative relationship between drought-responsive transcriptional regulation and potassium feeding. Similar outcomes were observed in Arabidopsis and maize, where potassium supplementation improved drought tolerance by upregulating the expression of stress-responsive transcription factors [2, 26, 31, 32].

Enhancing barley’s tolerance to drought stress is a practical application of these results. As a component of the food supply and a modulator of stress signals, potassium acetate activates transcriptional pathways that increase a plant’s resistance to water deprivation. Potassium acetate was associated with higher DREB transcript abundance in treated plants, as assessed by semi-quantitative RT-PCR. Potassium acetate influenced growth and DREB transcript levels under regulated, non-stress settings. Additional drought assessments are necessary to ascertain if these responses result in enhanced stress tolerance.

The findings indicate that, at appropriate concentrations, potassium acetate functions as a bio-stimulant, promoting barley development and triggering vital cellular pathways that respond to stress. Overdosing may cause stress, which emphasizes the need for precise dosage optimization in agricultural applications.

## Conclusion

The current study shows that potassium acetate has a clear concentration-dependent effect on barley (*Hordeum vulgare* L., cv. Giza 134), combining morphological, physiological, and molecular responses into a coherent stress-adaptive framework in Hordeum vulgare. Moderate dosages (1–2 mM) considerably increased germination, biomass accumulation, leaf growth, photosynthetic pigment content (chlorophyll b, carotenoids, and xanthophylls), and total protein levels, demonstrating its effectiveness as a bio-stimulant. Multivariate studies (heatmaps, clustering, and PCA) consistently revealed 2 mM as the best treatment for overall results. Molecular analyses found modest polymorphism, with ISSR markers being more sensitive than RAPD, indicating treatment-associated genomic variation but no indication of heritable instability. Importantly, sequencing verified the presence of a conserved DREB gene, and expression profiling revealed considerable increase at 2–3 mM potassium acetate, indicating that dietary manipulation activates stress-responsive transcriptional pathways. Higher concentrations (≥ 3 mM) caused partial growth inhibition and metabolic changes, indicating a stress threshold. Collectively, these findings suggest potassium acetate as a precision nutrition strategy capable of increasing barley growth and triggering drought-related molecular responses, while underlining the importance of dose optimization for long-term agricultural applications.

Potassium acetate may function as a bio-stimulant at low concentrations by improving pigment production and light collection ability, enhancing the body’s ability to combat free radicals, and promoting protein synthesis, which may be linked to enzyme activity or stress responses. However, higher quantities (≥ 3 mM) may produce metabolic stress; therefore, careful adjustments are necessary in agricultural contexts.

Finally, combining sequencing, phylogenetic analysis, and expression profiling provides a comprehensive understanding of DREB gene activity in potassium acetate-treated barley. These findings contribute to the overall framework for enhancing abiotic stress resistance through precision nutrition control and molecular breeding approaches.

## Supplementary Information

Supplementary Material 1.

Supplementary Material 2.

Supplementary Material 3.

## Data Availability

The datasets generated and/or analyzed during the current study are available in the [NCBI database, with accession number: PZ127018] at the following link [https://www.ncbi.nlm.nih.gov/WebSub/?form=final&sid=3061508&tool=genbank
