# Supplementary material for: Integrated analysis of potassium acetate effects on barley: growth, physiological traits, genetic diversity, and DREB gene regulation
Source: BMC Plant Biol. 2026 Apr 2;26:644. doi: 10.1186/s12870-026-08586-8 (PMC13064094; doi:10.1186/s12870-026-08586-8)
Supplement: Supplementary file 1 — Supplementary Material 1. [file 12870_2026_8586_MOESM1_ESM.docx]

Supplementary file

Supplementary table 1 The EC and pH of soil before and after treatments with pot. acetate

| Concentration | EC (dS m⁻¹) | | pH | |
| --- | --- | --- | --- | --- |
|  | Before treatment | After treatment | Before treatment | After treatment |
| Control (0 mM) | 0.25 | 0.28 | 8.00 | 7.95 |
| 1 mM | 0.25 | 0.32 | 8.00 | 7.96 |
| 2 mM | 0.25 | 0.40 | 8.00 | 7.93 |
| 3 mM | 0.25 | 0.55 | 8.00 | 7.88 |
| 4 mM | 0.25 | 0.70 | 8.00 | 7.82 |
